# The Spread of Fecally Transmitted Parasites in Socially-Structured Populations

**DOI:** 10.1371/journal.pone.0021677

**Published:** 2011-06-30

**Authors:** Charles L. Nunn, Peter H. Thrall, Fabian H. Leendertz, Christophe Boesch

**Affiliations:** 1 Department of Human Evolutionary Biology, Harvard University, Cambridge, Massachusetts, United States of America; 2 CSIRO Plant Industry, Canberra, Australia; 3 Research Group Emerging Zoonoses, Robert Koch-Institute, Berlin, Germany; 4 Max Planck Institute for Evolutionary Anthropology, Leipzig, Germany; University of Western Ontario, Canada

## Abstract

Mammals are infected by a wide array of gastrointestinal parasites, including parasites that also infect humans and domesticated animals. Many of these parasites are acquired through contact with infectious stages present in soil, feces or vegetation, suggesting that ranging behavior will have a major impact on their spread. We developed an individual-based spatial simulation model to investigate how range use intensity, home range overlap, and defecation rate impact the spread of fecally transmitted parasites in a population composed of social groups (i.e., a socially structured population). We also investigated the effects of epidemiological parameters involving host and parasite mortality rates, transmissibility, disease–related mortality, and group size. The model was spatially explicit and involved the spillover of a gastrointestinal parasite from a reservoir population along the edge of a simulated reserve, which was designed to mimic the introduction pathogens into protected areas. Animals ranged randomly within a “core” area, with biased movement toward the range center when outside the core. We systematically varied model parameters using a Latin hypercube sampling design. Analyses of simulation output revealed a strong positive association between range use intensity and the prevalence of infection. Moreover, the effects of range use intensity were similar in magnitude to effects of group size, mortality rates, and the per-contact probability of transmission. Defecation rate covaried positively with gastrointestinal parasite prevalence. Greater home range overlap had no positive effects on prevalence, with a smaller core resulting in less range overlap yet more intensive use of the home range and higher prevalence. Collectively, our results reveal that parasites with fecal-oral transmission spread effectively in socially structured populations. Future application should focus on parameterizing the model with empirically derived ranging behavior for different species or populations and data on transmission characteristics of different infectious organisms.

## Introduction

Mammals are host to a wide diversity of infectious agents [1], [2]. Many of these parasites and pathogens are gastrointestinal and spread through fecal-oral transmission routes which involves fecal contamination of the soil, food items or other substrates and subsequent consumption of infectious stages of the parasite by other hosts. Examples of fecally transmitted micro- and macroparasitic organisms found in wild mammals include protozoa such as *Giardia* and *Cryptosporidium* [3], [4], intestinal nematodes such as *Ascaris*, *Enterobius* and their close relatives [5], many species of fungi [6], bacteria such as pathogenic *Escherichia coli* [7], and viruses such as adenoviruses [8]. In wild primates, the prevalence of gastrointestinal macroparasites can exceed 50% [9], [10]. A variety of gastrointestinal infectious agents are also well known in human populations, including Norwalk virus, pathogenic *E. coli*, cholera, and *Cryptosporidium*. Many of these infectious organisms – hereafter also referred to simply as parasites – are harmful to wild animals, for example by increasing mortality and reducing fecundity [11], [12], [13], [14].

Despite growing knowledge of the parasites that cause wildlife infections, the dynamics of fecally transmitted infectious agents in natural animal populations are still not well understood. An individual mammalian host harboring a gastrointestinal parasite may shed large numbers of infectious agents to the environment, potentially infecting other animals in close proximity or those that come into contact with excreted material at a later time. This contact may occur, for example, when individuals from different groups overlap at food or water resources (i.e., home range overlap), suggesting that heterogeneity in resource distribution could play a major role in the dynamics and persistence of fecally transmitted infectious agents. In addition, some gut pathogens such as cholera result in diarrhea, which could benefit the pathogen by disseminating infectious stages more widely, especially when host movement is not impaired or when fecal material can contaminate water sources. Thus, a number of important epidemiological questions arise concerning interactions among factors involving host sociality, ranging patterns and parasite transmission mechanisms [15], [16], [17].

Parasites are of increasing concern in the conservation of biodiversity [18], [19], [20], [21], including the decline of animals that typically live in socially structured populations, such as primates [22], [23], [24], [25]. At an applied level, understanding the dynamics of infectious disease in relation to anthropogenic impacts and population structure – and how these might influence subsequent ecological and evolutionary trajectories of parasites in terms of virulence and transmissibility – is critical for making informed conservation management decisions [21], [26]. Of relevance in this context, domesticated animals and humans along habitat edges may introduce new parasites into wild populations, which can then spread based on social, ecological and infection characteristics of the system [7], [27]. Given that wild animals, domesticated animals and humans often overlap along the edges of nature reserves, it is critically important to improve our understanding of the ecological factors that enable some parasites to penetrate and persist in host populations of wild animals [20], [28], [29].

Several studies have investigated how range use behavior might influence the spread of fecally transmitted parasites. For example, territoriality could reduce home range overlap and contact between groups, resulting in fewer opportunities for the spread of parasites [the “territoriality benefits” hypothesis, 30]. Conversely, more intensive use of a home range could increase exposure to fecal material in the home range, resulting in higher levels of infection [the “fecal exposure” hypothesis, 16]. In a comparative test of parasite richness aimed at investigating these possibilities, Nunn and Dokey [15] found that helminth richness covaried positively with the intensity of range use in primates, thus providing support for the fecal exposure hypothesis over the territoriality benefits hypothesis. They also investigated whether home range overlap influenced parasite diversity across host species, but found no significant effects. In ungulates, Ezenwa [16] found that territorial species have higher prevalence of parasitic nematodes (strongyles) than non-territorial species and, among gregarious hosts, territorial species were found to have higher richness than non-territorial species. Similarly, amongst two groups of mantled howler monkeys (*Alouatta palliata*), Stoner [31] found that parasitism was higher in a group that used a narrow forest corridor between two blocks of forest (rather than a more cohesive block of forest for the other group). The more intensive use of habitat in the corridor was one of several factors that may have increased parasitism in the more heavily infected group [see also 28].

Here, we developed an individual-based model to investigate how social, ecological and parasitological factors influence the spread of fecally transmitted infectious agents in socially structured populations (i.e., where individuals live in spatially distributed social groups and disperse among groups). In socially structured populations, parasites face a major challenge in spreading from one group to another; groups are in effect “islands” for parasites, and this effect might be strengthened if territorial behavior and social structure further restrict movement of parasites [29], [32], [33], [34]. For fecally-transmitted parasites, three major routes of group-to-group transmission seem most likely: through movement of infected individuals among groups, resulting in the introduction of the parasite to the home range of a new group; through shared resources and resulting home range overlap among groups; and through direct social interactions between groups, including mating and territorial interactions. We focused on the first two of these mechanisms by investigating whether the rate of movement between groups (dispersal) or range overlap has a bigger impact on the spread of parasites in socially structured populations. We did not explicitly model intergroup encounters, and thus we do not directly consider how territoriality could reduce infection risk by limiting home range overlap or social interactions. To investigate the “fecal exposure” hypothesis, we varied the intensity of home range use (i.e., day range relative to home range).

In addition to dispersal and ranging, our model incorporated several other factors expected to play key roles in gastrointestinal parasite dynamics. Group size may be important if larger groups produce more fecal contamination per unit area of the environment, both within the group's home range (causing more individuals to become infected) and outside the range (causing other groups to become infected). Some gut parasites may increase fecal output (e.g., diarrhea), which could lead to increased spread of infectious agents among individuals in social groups. To study whether increasing fecal output might influence infectious disease dynamics, we varied the rate at which infected individuals defecated. In addition, a variety of standard epidemiological parameters should influence the dynamics of gastrointestinal parasites. Thus, a higher background mortality rate or death rate due to disease should reduce the ability of a parasite to become established in a host population. Similarly, the spread of infectious organisms will be enhanced by a higher transmission rate and longer infectious period in the soil or in the host (provided that the benefits of longer infectious periods are not offset by higher disease-related mortality). A longer latent period in the soil, however, may reduce parasitism rates, because with longer latency, groups will on average be farther from the site of defecation when the parasites become infectious.

Several studies have documented the potential for infections to spread from humans and their domesticated animals into wildlife [e.g., nonhuman primates, 7,27,35,36]. Thus, we explicitly investigated the conservation implications for fecally transmitted parasites by modeling the introduction of infectious organisms along one edge of a simulated “reserve” and quantifying the spread of infections across the reserve.

In our simulation model, individual hosts are part of social groups that range on a landscape composed of 81 distinct social groups, where individuals of the same social group range as a cohesive unit on the landscape. The hosts are exposed to fecal contamination from a hypothetical population of domesticated animals that border one edge of the landscape; the possibility for infection occurs when individuals come into contact with feces from this domesticated animal reservoir. Newly infected individuals then spread the infection to other individuals in their groups, and to individuals in different groups through dispersal or in areas of home range overlap. We use the model to evaluate the relative importance of social, ecological and parasitological factors likely to influence the spread of fecally transmitted infections.

## Results

**Figure 1:**
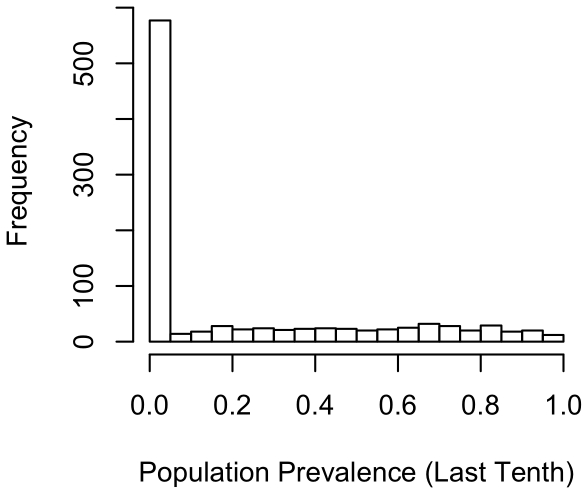
Proportion of the population infected. The histogram shows the frequency in which particular proportions of the population were infected. Results are based on average prevalence over the last one-tenth of time steps (730 steps in total) across 1000 simulations.

We conducted 1000 simulations that varied the 12 parameters according to the minima and maxima shown in Table 1. As output, we focused on prevalence of infection with the parasite and population loss at various points in the simulation (prevalence related terminology is summarized in Table 2). Both population dynamics and prevalence of infection varied greatly across simulation runs, with mean population prevalence ranging from 0 to 98.7% (calculated over the last one-tenth of time steps for each of 1000 simulations, at which point infection dynamics had stabilized). In most simulations, however, population prevalence was low (Figure 1, median prevalence  = 0.4%, mean  = 22.4% over the last one-tenth of time steps).

**Table 1:** Simulation parameters and range of values used (Latin Hypercube Sample).

Parameter	Units	Minimum	Maximum	Midpoint	Upper Quartile
Group size (*g*)	Individuals	4	40	22	31
Mortality rate^1^ (*m_b_*)	Probability per time step (day)	0.000055	0.0055	0.0028	0.0014
Disease mortality (*m_d_*)	Probability per time step (day)	1	100	50.5	25.75
Day range^2^ (*D*)	Range matrix grid cells	2	30	Variable	Variable
Core area^3^ (*c*)	Proportion of a group's range matrix	0	0.5	0.25	0.375
Latency – soil^4^ (*b_s_*)	Time steps (days)	1	15	8	11.5
Infectious – soil^4^ (*f_s_*)	Time steps (days)	1	50	25.5	37.75
Latency – host^4^ (*b_h_*)	Time steps (days)	3	14	8.5	11.25
Infectious – host^4^ (*f_h_*)	Time steps (days)	4	365	184.5	274.75
Defecation rate^5^ (*d*)	Probability per time step (day)	0.5	3	1.75	2.375
Transmission (*β*)	Probability per time step (day)	0.00001	0.001	0.00051	0.00075
Dispersal rate (*i*)	Probability per time step (day)	0.001	0.01	0.0055	0.0078

1 Based on life span range of 0.5–50 years and time step of one day.

2 Number of movement steps per simulated time step. Range was based on values of the D-index from Mitani and Rodman [37], i.e. 0.2 to 3 when converted to the D-index.

3 Rounded down to increments of 0, 0.1, 0.2, 0.3, and 0.4.

4 Integer values.

5 For infected hosts only, and used as a rate per day calculated as *d* / *D*.

**Table 2:** Output Measures.

Measure	Description	Timeframe Used for Calculation
Population prevalence	Prevalence based on all individuals in the population	Mean or median over the last 730 time steps (last 1/10 of the simulation) or in final time step
Total population loss	Change in population size as a percentage of the starting population	Time step 1 to time step 7300
Group prevalence	Prevalence of individuals in groups, averaged across groups, and useful for assessing what proportion of groups are infected	Last time step
Group prevalence at edges	Prevalence of infection among individuals along particular segments of the reserve, measured relative to the spillover population	Last time step
Maximum prevalence	Maximum recorded population prevalence over the course of the simulation	Time step 1 to time step 7300
Number of groups infected	Number of groups that were infected when a simulation ended	Time step 7300

**Figure 2:**
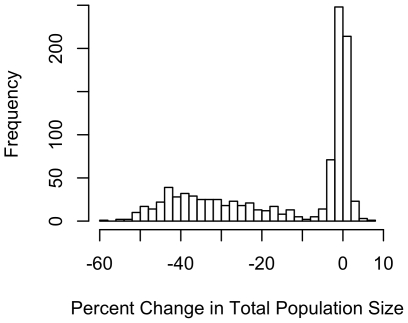
Proportion of the population lost. The histogram shows a bimodal distribution of changes in population size. In most simulations, population changes were slightly negative or positive, reflecting stochastic variation related to births and deaths (indicated by the tall bars around zero). In a sizable number of simulations, however, population losses exceeded 10%.

Total population loss over the simulation showed a bimodal distribution (Figure 2). For many parameter combinations, changes in population size were characterized by only slight losses or gains (expected due to the stochastic effects of births and deaths, shown as the highest peak around zero in Figure 2). However, 41.9% of the simulations resulted in losses of more than 10% of the population. Maximum population loss was 58.8%.

Along the edge where infections were introduced, mean group prevalence was 22.8% (maximum of 98.9%), and group prevalence was greater than zero in 72.7% of simulations. A similar pattern was found at the far edge of the reserve (i.e., furthest from the source of new infections), with mean group prevalence of 21.5% (maximum  = 98.7%). At the far edge, however, fewer simulations showed group prevalence greater than zero (43.3% of simulations), probably due to the continual introduction of parasites at the near edge resulting in a constant inflow of infections.

In a general linear model of the 1000 simulations using the Latin hypercube sample of input parameters, we found that average population prevalence (recorded at the last time step of simulations) was best explained by group size, parasite infectious period in the soil, and transmission probability (Table 3, *R^2^* = 0.60, *F_12,987_* = 127.4). Prevalence also increased with day range and the rate of defecation. Increases in both intrinsic mortality and disease-related mortality had negative effects on prevalence. Importantly, the size of the core area had an effect on population prevalence that was opposite to predictions of the territory benefit hypothesis. A smaller core area reduced home range overlap, but tended to increase population prevalence (Table 3), probably because a smaller core area resulted in greater re-use of cells in the core area. Thus, counter to expectations under the territory benefits hypothesis (and across a wide range of parameter values), reduced overlap with neighboring groups failed to result in lower prevalence at the population level.

**Table 3:** General linear modeling of average population prevalence.

Predictor	Estimate	t-statistic
Intercept	0.224	36.4
Group size (*g*)	0.105	17.0
Infectious – soil (*f_s_*)	0.105	17.0
Transmission (*β*)	0.100	16.1
Day range (*D*)	0.087	14.1
Disease mortality (*m_d_*)	−0.083	−13.4
Mortality rate (*m_b_*)	−0.066	−10.7
Defecation rate (*d*)	0.062	10.0
Smaller core area (*c*)^1^	0.051	8.19
Latency in host (*b_h_*)	0.030	4.77
Infectious – host (*f_h_*)	0.018	2.98
Dispersal rate (*i*)	0.006	1.00
Latency in soil (*b_s_*)	−0.003	−0.471

1 In the model, core area was parameterized as the difference from the edge of a ranging matrix to the edge of the core area. Thus, higher values of this difference indicate a smaller core area.

Another finding of interest is that dispersal rate did not have strong effects on population prevalence (Table 3). One possibility is that dispersal (and possibly home range overlap) has a greater impact on pathogen spread early in an epidemic, as compared to effects on prevalence when dynamics reached a steady state. We therefore examined population prevalence at an earlier stage in the simulations, over the first 1/10 of the simulation run (time steps 1 to 730, representing units of single days and thus equivalent to 2 years of transmission dynamics). Average population prevalence was 9.2% during this initial phase, which as expected was much less than average prevalence of 22.4% at the end of the simulation. A GLM of the predictors of population prevalence early in the simulations accounted for 52% of the variation in prevalence. Relative to the results at the end of the simulation (see Table 3), the standardized coefficient for the rate of dispersal increased three-fold (coefficient = 0.018, t_987_ = 4.84), while the effect of core area became substantially weaker but remained positive (coefficient = 0.008, t_987_ = 2.27, indicating that less overlap increases prevalence). These findings suggest that shortly after introduction, the rate of dispersal influenced the rate at which a gastrointestinal parasite spreads through a host population. Other results were similar to those presented in Table 3, with group size, infectious period in the soil, transmission probability and day range having the largest effects on prevalence (although with smaller standardized coefficients on average).

We also generated linear models to examine the predictors of maximum prevalence, group prevalence, number of groups infected, and population loss due to infectious disease. These analyses produced remarkably similar results, with the ranking of effects identical (or nearly so) to the results in Table 3 (see Supporting Information Tables S1 to S4). Of particular interest for conservation effort is population loss, with population loss increasing with increases in the following key variables: infectious period in the soil, group size, probability of transmission, and day range (see Table S4). A higher mortality rate (and higher disease related mortality) tended to depress the degree to which populations declined due to disease.

**Figure 3:**
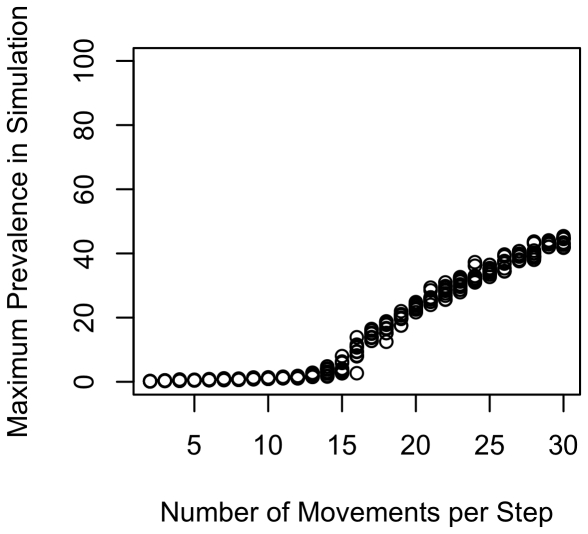
Maximum population prevalence in relation to day range: midpoint values. Plot shows how maximum prevalence increases with day range (movements per time step) when using the midpoint of the range of values in Table 1. Day range is the number of steps that a group moved on the ranging matrix per time step. Given a home range diameter of 10, values of the D-index can be obtained by dividing number of range movements by 10. Prevalence was taken as the maximum recorded prevalence across each simulation. Use of averages (rather than maxima) produced similar patterns.

**Figure 4:**
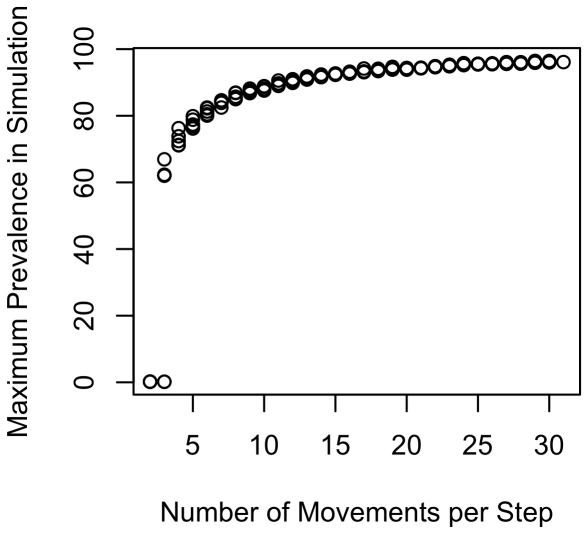
Maximum population prevalence in relation to day range: “upper” values. Plot shows how maximum prevalence increases with day range (movements per time step) when using the upper quartile of the range of values, where upper refers to the direction for the parameter that would be expected to increase prevalence. Day range is the number of steps that a group moved on the ranging matrix per time step. Given a home range diameter of 10, values of the D-index can be obtained by dividing number of range movements by 10. Prevalence was taken as the maximum recorded prevalence across each simulation. Use of averages (rather than maxima) produced similar patterns.

To illustrate the effects of ranging intensity, we re-ran the simulations holding all parameters constant except for day range, which we varied from 2 to 30. Setting all other variables to their midpoint values (Table 1), we found a positive association between ranging intensity and parasite prevalence (Figure 3 shows results for maximum recorded prevalence). In addition, the plot reveals a clear threshold around 12 movements per time step, with the infectious disease generally unable to persist at lower movement rates. However, the average population prevalence at the end of these simulations was fairly low (12.6%). We repeated the analysis with the upper quartile of values (or lower quartile for variables that show a negative association with prevalence, Table 3). We again found a strong association between ranging intensity and prevalence (Figure 4 for maximum prevalence), with much higher average population prevalence, as expected, at the end of simulations (84.2%).

**Figure 5:**
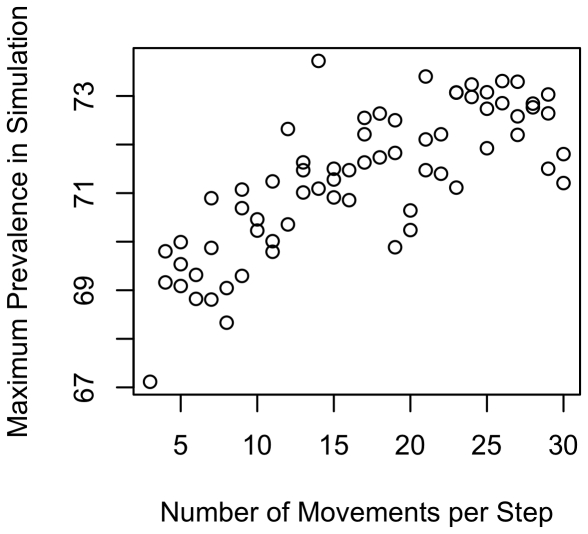
Maximum prevalence and movement when holding infection risk constant. Plot shows how maximum prevalence covaries with day range using the alternative ranging model. In this model, groups have the same number of opportunities for infection, regardless of day range.

Our results may be sensitive to the underlying ranging model that we used. To investigate this possibility, we implemented a different model of exposure in the home range for a focused set of parameters. Specifically, we altered the model to hold constant the number of exposure steps, as described in the Methods. Holding other variables constant at their midpoints, we found that day range had a positive effect on prevalence (Figure 5), albeit a weaker overall effect than in the general model (cf. Figures 3 and 4).

## Discussion

The general results from our model suggest that gastrointestinal parasites can be of significant conservation concern in socially structured populations of wild hosts by exhibiting high prevalence, causing significant population declines, and spreading effectively from one side of a simulated reserve to the other side across multiple home ranges. Although disease-related mortality should slow the spread of infectious agents, for the environmentally transmitted parasites in our simulated populations, it appears that even virulent parasites can spread widely. Partly this reflects the buildup of material in the soil that can remain infectious for many time steps, and partly it reflects that newly susceptible individuals are born into the population in a density-dependent manner.

Social groups represent biological islands for infectious disease, and thus exclusive use of a home range (i.e. reducing among-group contacts) might be expected to reduce the risk of parasitism [30]. Previous work in primates and ungulates suggests, however, that territoriality and its correlates, such as higher intensity of range use, increase the risk of infection with fecally transmitted parasites [15], [16]. One explanation for this effect is that territoriality tends to result in more intensive use of a home range [37], resulting in higher rates of re-infection. An alternative explanation is that territoriality and ranging are costly, for example in terms of physical effort and risk associated with defending the territory or elevated levels of testosterone or cortisol [16]. Thus, individuals who are defending a territory may be more susceptible to infectious disease. Similarly, parasites might spread among individuals in different groups through physical contact during inter-group encounters in a more territorial species.

Our model allowed us to assess whether greater exposure to parasites in the soil – generated from more intensive ranging – results in higher levels of infection at the group and population levels. We found strong evidence for greater range use as a driver of higher prevalence, with day range exhibiting effects that were similar to those found for fundamental epidemiological parameters involving transmission rate, mortality rate, and a combination of population size and contact rate (i.e., group size). Conversely, greater home range overlap appeared to have no effect on the spread of gastrointestinal parasites; overlap actually resulted in lower levels of infection (rather than the expected positive effect). This effect occurred because groups with greater overlap used their own core areas less intensively, suggesting again that intensity of range use is the primary ranging parameter that impacts prevalence. Similarly, rates of dispersal appeared to be important only during the initial spread of an infectious disease. We also investigated whether higher defecation rate in infected individuals impacts transmission dynamics. As expected, defecation rate had a significant positive impact on overall prevalence. However, compared to the effects of other parameters, such as mortality rates, defecation rate was less important (see Table 3 for standardized regression coefficients).

In the simulation model, we assumed that animals move in a random walk within their core ranges; outside the core, they moved in a biased random walk, with a tendency to return to their core area. Such a movement pattern could lead to higher rates of infection, given that animals are more likely to cover the same ground under a random walk when compared, for example, to animals exhibiting other movement patterns [e.g., 38]. Indeed, we expect that under more realistic models of ranging, latency periods in the soil might have greater impacts on the spread of parasites because animals might be less likely to encounter feces shortly after their deposition. In addition, larger social groups may require larger ranges [39], [40], which could reduce exposure to infectious stages in the soil and reduce disease risk. Individuals also could have spatial memory of resources and environmental risks, which may impact ranging patterns and thus patterns of infection [e.g., 41]. An important area for future research is to build stronger theoretical linkage between the risk of fecally-transmitted parasites and empirically derived patterns of ranging behavior and social interactions [e.g., in primates, 42,43].

We also assumed that ranging behaviors are independent of infection levels in the group. However, it is possible that groups with higher levels of infection might have shorter day ranges, for example if infected individuals show more sickness behaviors, such as resting [44]. Although not formally modeled here, our results suggest that disease-related reduction in ranging would reduce the spread of infection in the population. This could be investigated in future empirical and theoretical research, and suggests that efforts to reduce ranging by infected groups (e.g., through provisioning) could lead to reduced levels of infection at the broader population level.

Our model explicitly considered the conservation impacts of an introduced gastrointestinal parasite. To do this, we modeled the continuous spillover from a reservoir host, such as domesticated animals or humans, along one edge of a reserve containing a wild host population sub-structured into a large number of social groups. We found that a fecally-transmitted parasites penetrated the population very readily, commonly reaching the far edge of the reserve. In addition, the introduced gastrointestinal parasite could cause significant mortality, with more than 40% of the simulations resulting in loss of 10% or more of the original population.

Highly pathogenic infectious diseases have attracted much recent attention, such as Ebola in African apes [24], [45], [46], [47]. Our model suggests, however, that in the context of conservation concerns, gastrointestinal pathogens could be as important as infectious agents that are transmitted by close contact or by vector. For example, higher disease-related mortality tended to slow the spread of infectious disease in our model, as expected given that this reduces the pool of infected individuals in the population [48], yet population declines due to disease can be great and increase with increasing infectious period in the soil, group size, probability of transmission, and day range length (see Supporting Information, Table S4). By comparison, a previous model of infectious disease dynamics involving a highly virulent introduced pathogenic infection, which was modeled after Ebola, found that the infectious agent rarely spread widely in the population and never caused extinction of the simulated host population [33]. Of course, high rates of spillover from a reservoir population could lead to severe population declines for a highly pathogenic infectious disease, and these risks should be monitored closely. Our model suggests that simultaneous with such monitoring, we should also be aware of infections with less immediate mortality effects in wild animal populations. In addition, the model serves as a call for more information on characteristics of parasites that infect wild animals, so that latency, transmissibility and disease-related death rates can be parameterized more effectively.

In terms of applications, our model provides several new insights for the control of gastrointestinal infections in spatially and socially structured host populations. First and foremost, it appears that once such parasites enter a population, they commonly spread throughout the range, often relatively quickly. Thus, in terms of measures aimed at prevention of initial invasion and spread, it is essential to prevent the initial introduction of gastrointestinal parasites from reservoir populations. A model like ours could be used to investigate the effects of ranging behavior by the reservoir population, or to examine approaches aimed at reducing habitat sharing between reservoir and wildlife populations. Second, we cannot count on territorial behavior to reduce the risk of infectious disease establishment in a wild host population. Infectious diseases appear to spread remarkably easily through dispersal and shared range use, with day range more important than actual measures of home range overlap. Lastly, rates of dispersal appear less important to the spread of parasites than range use, but once dispersal of an infected individual into a new group occurs, infectious material can build up in the soil of the new group and result in new infections. Thus, it may be important to constrain host movements, both in terms of habitat sharing and dispersal, especially during early stages of infectious disease spread (i.e., while infection is spatially limited to a small number of social groups). However, this may only be possible for intensively managed wildlife such as those living in game ranches.

In summary, our study provides new insights into the role of ranging behavior on the spread of gastrointestinal parasites. While previous comparative and field studies have found such links, they were unable to establish that these links were caused by more intensive ranging, or by alternative mechanisms involving territoriality, such as increased susceptibility from stressors related to territorial encounters, or exposure to parasites at territorial boundaries. Our model demonstrates that ranging behavior is likely to have strong effects on parasitism that are equivalent in magnitude to other well-established epidemiological factors. Moreover, by including a spillover host, our model demonstrates the importance of gastrointestinal parasites for conservation of biodiversity. Collectively, our results highlight the need for renewed attention to reducing the flow of infections into wildlife populations, and for greater empirical effort to investigate whether ranging and other behaviors increase the spread of these parasites into wildlife.

## Methods

### Basic Simulation Structure

The goal of the model was to investigate the social, ecological and epidemiological parameters that influence the spread of fecally transmitted parasites in socially structured populations, specifically in the context of spillover from a neighboring population of animals on the edge of the habitat (such as domesticated animals or humans). The model was designed to simulate the spatial movement of groups of individuals relative to a “core area” of the home range, and the movement of individuals between different social groups through dispersal.

**Figure 6:**
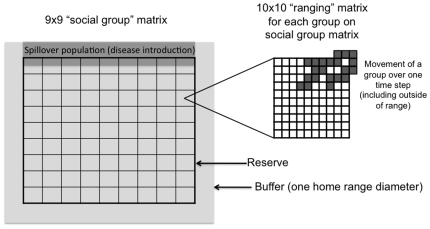
Social group, ranging and spillover areas. Social groups are arranged on the landscape and identified by the *social group lattice*, which was a 9×9 lattice in all simulations presented here (n = 81 social groups). Social groups range within the *ranging lattice*, which is a 10×10 lattice for each of the 81 social groups and contains a core area (see Figure 2). The 81 10×10 ranging lattices constitute the *reserve*. Around this reserve, a further 10 cell *buffer* occurs, producing a total potential ranging area of 110×110. Along the top edge of the buffer and reserve, a *spillover population* exists; it penetrates the reserve within 5 cells, thus overlapping with the uppermost social groups of the *focal population*.

Simulations took place on a 9×9 square lattice (*social group matrix*), within which smaller square lattices of cells (*ranging matrix*) were designated that reflect a *home range* (10×10 cells) for each group on the social group matrix; collectively, the area of the range lattice that includes social groups for the focal host species was referred to as the *reserve* (Figure 6). Within each home range a *core area* was further defined as the percentage of cells away from the edge of the home range that the animals prefer to use. When this parameter equaled zero, the core area and the home range coincided (10×10, i.e. 100 cells); when the parameter equaled 0.1, the core area represented the inner 64 cells (8×8); and when this parameter equaled its maximum of 0.5, the core area was a point in the center of the range (0×0 core area, and thus agents tightly used the center of the range). Given that animals prefer to range in their core area but can move outside of it (see below), a smaller core area resulted in less home range overlap among groups, which was confirmed using data on group location recorded during the simulations.

Around the range lattice we then added a further 10 cells, which is equivalent to one home range. This *buffer* enabled groups of the *focal population* to range outside the reserve, and for a second species (the *spillover population*) to contaminate one edge of the reserve with infected feces. Parasites were introduced to the focal population from the spillover population (e.g., infected cattle), which in our simulations always ranged along the upper edge of the reserve and penetrated one-half of the home range of the nearest focal population by five cells (Figure 6). Spillover infections occurred at the rate of 10 infected feces scattered randomly in this area in each time step of the simulation.

Feces containing infectious stages of parasites accumulated in the range lattice and, following a *soil latency period* on the ground, were potentially infectious during a *soil infectious period* to individual hosts in the focal population. We thus took into account that parasites exhibit a latency period and mortality during the soil stage (i.e., they were not continuously infectious).

Individual hosts were associated with one of the 81 groups in the social structure lattice, and each group had a location in the range lattice that was typically, but not always, in the designated home range of that particular group (see “Group Social Behavior and Population Dynamics” below). Individuals were further characterized by their infection status, including number of days in a defined *host latency period* (i.e., exposed but not yet infectious) and, following host latency, number of days in a *host infectious period*. During the host infectious period, feces were produced that are infectious to other individuals after a soil latency period. The defecation rate was defined as number per day rather than per movement step in a day, and thus was comparable across simulations with different day ranges. Infection occurred with transmission probability *ß* for each infectious pile in the ranging grid cell, and the probability of infection was calculated for each movement step and, in a subset of simulations, holding this constant per day. We assumed that after clearing the infection, individuals have no immunity to the infectious agent and thus were susceptible to re-infection (i.e., a susceptible-exposed-infected-susceptible model). While infected, however, individuals could not become infected with another parasite; thus, the individual had to move through the infectious periods to be re-infected. Some gastrointestinal infectious agents may elicit varying degrees of immunity, but we did not consider this possibility in our current model.

We ran each simulation for 7300 time steps, which were in units of one day and thus equivalent to 20 years of infection dynamics. In initial runs under a wide variety of parameter settings, we determined that the simulation reached a steady state well before time step 7300. Specifically, we recorded key statistics, such as prevalence (see Table 2), across 10 blocks of 730 time steps each (corresponding to 2 year periods). We then confirmed empirically that prevalence had stabilized by the last 1/10 of the simulation.

### Model Parameterization and Exploration

For each simulation run, groups of individuals were formed based on user-specified values for group size by drawing random numbers from a Poisson distribution. All groups had at least two individuals, and all individuals in the population were initially uninfected. Groups were then assigned a random location on the range matrix. Deaths, births and dispersal of individuals will tend to cause the initial social group structure to drift over a simulation run, especially when simulations are run for many time steps. To help maintain initial demographic conditions, we retained a matrix of the initial numbers of males and females in each group. This *initiating matrix* was used to stochastically adjust probabilities associated with demographic parameters (birth and dispersal) to help maintain initial conditions for each group throughout a simulation run (see below).

To explore how different parameters influence disease dynamics, we undertook multivariate analyses using random sampling. Random sampling was conducted using Latin hypercube sampling, which is a type of stratified Monte Carlo sampling that has been used in epidemiological modeling and is more efficient in this context than random sampling regimes or those that include all possible parameter values [49], [50], [51], [52]. Twelve parameters were varied across uniform (flat) distributions in the Latin hypercube sample: group size, transmission probability, background mortality, disease-related mortality, rate of dispersal, defecation rate, day range, core area, soil latency period, host latency period, and host infectious period. Table 1 summarizes the parameters that we investigated, along with the ranges of variation that were sampled for each parameter. Parameters that required integer or discrete values for the model (e.g., host infectious period) were represented as continuously varying traits in the Latin hypercube sample and then averaged appropriately. Using this approach, we generated 1000 Latin hypercube samples reflecting the range of variation in Table 1 (i.e., 1000 simulations).

In addition to the Latin Hypercube sample, we undertook an additional set of analyses to investigate how day range influenced prevalence while holding other parameters constant. We conducted these analyses using the midpoint of values from the Latin Hypercube sample, and then repeated the process using the upper or lower quartile as the value (selecting upper or lower quartile values to produce higher expected prevalence, based on the results from the Latin hypercube sample and epidemiological theory). The values used are given in Table 1.

### Group Social Behavior And Population Dynamics

Model dynamics proceeded in discrete time steps, which represent single days in the lives of the simulated agents. In each time step four processes took place sequentially: (1) ranging and possible infection of hosts due to exposure to feces in the ranging matrix, (2) deaths due to stochastic factors and infection, (3) stochastic dispersal of individuals to neighboring groups, and (4) stochastic births to replace individuals lost to disease or other factors. These processes are explained in further detail below.

**Figure 7:**
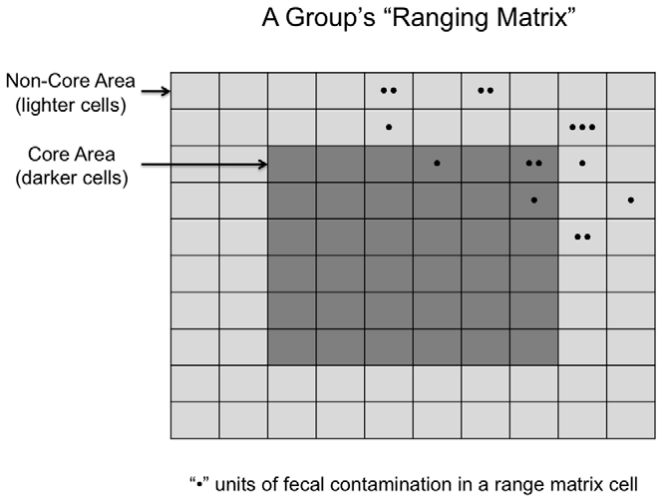
Core area and fecal contamination. Within each 10×10 ranging area per group, a *core area* is defined as a proportion of the range and centered in it. This core is identified as a certain number of cells in from the range. In this case, the core area is 2 cells from the edge, giving a 6×6 core area. Groups range with a random walk within the core, and exhibit a tendency to move toward the core when outside of it, where the bias toward the core is a function of how far the group is currently away from the core. This figure further shows the build-up of infectious material (fecal contamination) within and outside the group's core area. An individual cell in the range matrix can have zero, one or multiple feces that harbor infection, and risk of infection increases with increasing fecal contamination.

Individuals moved in their home ranges (i.e., the ranging matrix) with other members of their social group. Groups ranged in a random walk within their *core areas* on the range matrix (Figure 7), and all members of a group moved as a cohesive unit in the same ranging matrix cell (i.e., groups are cohesive). Core areas were centered inside a group's designated home range, and thus did not overlap with other groups' core areas.

Groups could range outside of their defined core areas, including into other groups' home ranges and core areas, but they did so with a “rubber-band” process that tended to pull them back towards the core area (and thus ranging is not a random walk when a group is outside the core area). More specifically, in a given time step, a random draw determined whether a group moved horizontally or vertically. Assuming that a vertical movement was selected, a group within its core area has an equal probability of moving either up or down, which is then determined by drawing a random number. Outside the core area, however, this decision to move up or down was biased by the vertical distance from the edge of the core area. Specifically, to the base probability of 1/2 for moving up or down, we added one to both numerator and denominator for each cell away from the core for the probability of moving back to the core. Thus, if the group was one cell “above” its core area, the probability of moving “down” on the next step became 2/3, if it was two cells away the probability was 3/4, if three cells away the probability was 4/5, and so on, asymptotically to a probability of 1. The same procedure was used for movements in the horizontal direction. Hence, the probability of movement toward the core area increased with distance outside the core area. All movements were independent of previous moves.

Two further constraints were placed on ranging behavior. First, groups were unable to move off the total matrix, which included the social group matrix plus the buffer zone equivalent to one home range diameter that surrounded the reserve (see Figure 6). Second, groups could not occupy a grid cell already occupied by another group in that step. When movement brought a group to a boundary or an already occupied cell, the ranging procedure was repeated up to 10 times, and if a suitable range cell was not located, the social group remained where it was for that time step.

The ranging component of the model has two key parameters: the core area affects the probability of overlap with other groups (relevant to the territory benefits hypothesis), while the day range impacts the intensity of range use (and thus exposure to parasites in the soil and relevant to the fecal exposure hypothesis). We consider each of these in turn.

A larger core area meant that groups tended to range closer to the boundary of their home ranges before the rubber band process biased movement back to the group's core area within its home range. In such cases, a given group could cross over into a neighboring group's range or into the buffer, including the area where the spillover population was located (Figure 6). Thus, a larger core area increased the probability that a group overlapped with the range of another group or the reservoir host. Conversely, a smaller core area (which was centered in the group's range) meant that groups were less likely to range outside of their home ranges, resulting in decreased home range overlap.

Range use intensity also was varied systematically. In primates and other mammals, researchers have used a measure known as the defensibility index (D-index) to measure range use intensity [37], and this measure was investigated in a recent comparative study of parasitism and primate ranging [15]. The D-index measures the intensity of range use by examining day journey length relative to home range size. Here, all groups had the same home range size; hence, the D-index was varied by simply changing the day range. In other words, greater range use intensity was equivalent to increased number of ranging movements per time step, as described above. We therefore refer to day range intensity simply as *day range* (*D*).

Following each movement to a new cell, three further stochastic processes took place in the following order. First, infected individuals defecated with probability *d* (adjusted for the number of movement steps per time step to make *d* comparable across simulation runs). The location of feces was recorded on the range lattice based on the location of the group, and following the soil latency period, they became infectious to individuals occupying that cell in future time steps within the soil infectious period. Uninfected individuals in the range cell were exposed to infectious fecal material and become infected with probability *β* per fecal pile in the cell. Finally, 10 feces per time step were placed randomly within the northernmost cells on the range lattice [i.e. within the area defined by coordinates (1,11), (15,11), (1,100), and (15,100), see Figure 6]. These ten fecal contaminations were assumed to come from the infected spillover population, and they underwent the same process of soil latency and infectious periods as described for parasites deposited by hosts in the focal population. We did not explicitly model the ranging behavior of the spillover population.

In our model, a larger day range corresponded to more opportunities for infection because each “movement step” during ranging (the day range) was associated with an opportunity for infection when the group was located on a ranging cell with infectious material. By doing this, we assumed that greater movement is equivalent to greater utilization of the habitat; thus, groups with larger day ranges used their habitat more intensively, resulting in more opportunities for infection as they moved. Instead of considering movement steps, infection could be based on the time available per day, and thus held constant across simulations with different day ranges. In this case, it is possible that by staying in the same grid cell, agents would be more exposed to existing parasites in that cell and might defecate, resulting in buildup of infectious material even when they are not moving. We therefore ran an additional set of simulations that kept the number of movement steps constant for each time step, with the probability of actual movement represented as a linear function of the day range. Averaged across time steps of a simulation, this procedure produced the user-defined day range, while holding time available for exposure constant across simulations.

The second step in the model dynamics involves disease-related and background mortality. Each individual experienced a baseline probability of death (*m_b_*), and infectious individuals had an additional source of mortality due to disease (*m_d_*), where *m_d_* was simply a multiplier of *m_b_* (range of values is given in Table 1). Infected individuals that died were removed from the simulation and could no longer infect other individuals.

The third step involved dispersal of individuals to neighboring groups with probability *m*. Individuals always moved to adjacent neighboring ranges, which were selected randomly. Dispersal was completed in one time step.

Lastly, births occurred for groups with at least one individual present. We recorded the initial population size and also the initial sizes of each group, and assigned a higher probability of birth if the current population size was less than the initial population size (and conversely, a lower probability if the current population was larger than its initial size). The baseline probability of birth (*b*) was set to equal the baseline probability of death (*m_b_*) when the current population size was equal to the initial population. When the current population departed from initial conditions, the probability of birth was set to *m_b_^f^*, where *f* is the current population size as a proportion of the initial population size. We calculated the number of births for populations as a random draw from the binomial distribution with probability *b^f^*, and then assigned births to groups. Groups that were smaller in the current time step relative to the initializing values, but that still had at least one individual in the group, were given a higher probability of receiving a birth. Specifically, they were twice as likely to be assigned a birth as other groups that matched or exceeded their group size at time step 1.

### Statistical Analyses of Model Output

We used general linear models to investigate how parameters from the Latin Hypercube sample influenced average prevalence, maximum prevalence, group prevalence, number of groups infected, and population loss due to disease (i.e., total number of individuals that die). The data were continuously varying, and we checked the normality of residuals to investigate the appropriateness of the statistical models. Because significance levels are sensitive to the sample size and here we are interested in relative effects, we avoided interpreting the findings based on frequentist statistical tests of null hypotheses, such as p-values. Instead, we standardized all the predictor variables prior to analysis by subtracting, for each datum, the mean of the data for that predictor and dividing by the standard deviation. We thus estimated standardized regression coefficients and interpreted larger coefficients as corresponding to larger effects. In addition, several of the variables were expressed in the Latin Hypercube sample as continuously varying, but effectively treated in the simulation as taking specific discrete values. Thus, for core area, we used values binned into increments of 0, 0.1, 0.2, 0.3, and 0.4, and we examined the actual number of expected defecations per day, which was normalized relative to the day range. Analyses were conducted in *R* [53].

In addition to analyses of the 1000 simulations in which we used the LHS of parameter values, we provide simple graphical output for data from simulations that varied the day range while holding other variables constant, including for the variant of the model in which opportunities for infection were held constant across different simulated day ranges.

## Supporting Information

Table S1 General linear model: predictors of maximum prevalence.
(DOC)

Table S2 General linear model: predictors of average group prevalence.
(DOC)

Table S3 General linear model: predictors of number of groups infected.
(DOC)

Table S4 General linear model: predictors of population decline due to disease.
(DOC)
