# Supplementary material for: The Spread of Fecally Transmitted Parasites in Socially-Structured Populations
Source: PLoS One. 2011 Jun 30;6(6):e21677. doi: 10.1371/journal.pone.0021677 (PMC3128086; doi:10.1371/journal.pone.0021677)
Supplement: Table S1 — General linear model: predictors of maximum prevalence. (DOC) [file pone.0021677.s001.doc]

Table S1. General linear modeling of maximum prevalence

| Predictor | Standardized Beta | t-statistic |
| --- | --- | --- |
| Intercept | 0.251 | 39.2 |
| Infectious – soil (*f_s_*) | 0.113 | 17.5 |
| Transmission (*β*) | 0.110 | 17.2 |
| Group size (*g*) | 0.108 | 16.7 |
| Day range (*D*) | 0.094 | 14.6 |
| Disease mortality (*m_d_*) | -0.087 | -13.5 |
| Mortality rate (*m_b_*) | -0.071 | -11.0 |
| Defecation rate (*d*) | 0.067 | 10.4 |
| Smaller core area (*c*) | 0.051 | 7.94 |
| Latency in host (*b_h_*) | 0.032 | 4.91 |
| Infectious in host (*f_h_*) | 0.020 | 3.07 |
| Dispersal rate (*i*) | 0.007 | 1.13 |
| Latency in soil (*b_s_*) | -0.004 | -0.673 |

R^2^=0.61, F_12,987_=133.3
