# Supplementary material for: The Spread of Fecally Transmitted Parasites in Socially-Structured Populations
Source: PLoS One. 2011 Jun 30;6(6):e21677. doi: 10.1371/journal.pone.0021677 (PMC3128086; doi:10.1371/journal.pone.0021677)
Supplement: Table S2 — General linear model: predictors of average group prevalence. (DOC) [file pone.0021677.s002.doc]

Table S2. General linear modeling of average group prevalence

| Predictor | Standardized Beta | t-statistic |
| --- | --- | --- |
| Intercept | 0.224 | 36.9 |
| Infectious – soil (*f_s_*) | 0.105 | 17.3 |
| Group size (*g*) | 0.105 | 17.2 |
| Transmission (*β*) | 0.100 | 16.4 |
| Day range (*D*) | 0.086 | 14.1 |
| Disease mortality (*m_d_*) | -0.082 | -13.4 |
| Mortality rate (*m_b_*) | -0.063 | -10.4 |
| Defecation rate (*d*) | 0.062 | 10.2 |
| Smaller core area (*c*) | 0.051 | 8.34 |
| Latency – host (*b_h_*) | 0.029 | 4.72 |
| Infectious – host (*f_h_*) | 0.018 | 2.93 |
| Dispersal rate (*i*) | 0.006 | 0.93 |
| Latency – soil (*b_s_*) | -0.003 | -0.44 |

R^2^=0.61, F_12,987_=129.5
