# Supplementary material for: The Spread of Fecally Transmitted Parasites in Socially-Structured Populations
Source: PLoS One. 2011 Jun 30;6(6):e21677. doi: 10.1371/journal.pone.0021677 (PMC3128086; doi:10.1371/journal.pone.0021677)
Supplement: Table S3 — General linear model: predictors of number of groups infected. (DOC) [file pone.0021677.s003.doc]

Table S3. General linear modeling of number of groups infected

| Predictor | Standardized Beta | t-statistic |
| --- | --- | --- |
| Intercept | 33.3 | 43.4 |
| Group size (*g*) | 14.9 | 19.3 |
| Infectious – soil (*f_s_*) | 12.9 | 16.8 |
| Transmission (*β*) | 12.2 | 15.9 |
| Day range (*D*) | 10.5 | 13.7 |
| Disease mortality (*m_d_*) | -8.30 | -10.8 |
| Mortality rate (*m_b_*) | -6.37 | -8.25 |
| Defecation rate (*d*) | 6.10 | 7.92 |
| Latency – host (*b_h_*) | 3.24 | 4.21 |
| Smaller core area (*c*) | 2.77 | 3.59 |
| Infectious – host (*f_h_*) | 2.26 | 2.94 |
| Dispersal rate (*i*) | 1.11 | 1.44 |
| Latency – soil (*b_s_*) | -0.071 | -0.09 |

R^2^=0.58, F_12,987_=114.9
