# Supplementary material for: The Spread of Fecally Transmitted Parasites in Socially-Structured Populations
Source: PLoS One. 2011 Jun 30;6(6):e21677. doi: 10.1371/journal.pone.0021677 (PMC3128086; doi:10.1371/journal.pone.0021677)
Supplement: Table S4 — General linear model: predictors of population decline due to disease. (DOC) [file pone.0021677.s004.doc]

Table S4. General linear modeling of population loss due to disease

| Predictor | Standardized Beta | t-statistic |
| --- | --- | --- |
| Intercept | 0.144 | 40.5 |
| Infectious in soil (*f_s_*) | 0.068 | 19.0 |
| Group size (*g*) | 0.066 | 18.4 |
| Transmission (*β*) | 0.064 | 18.1 |
| Day range (*D*) | 0.057 | 15.9 |
| Mortality rate (*m_b_*) | -0.037 | -10.3 |
| Defecation rate (*d*) | 0.035 | 9.74 |
| Smaller core area (*c*) | 0.023 | 6.36 |
| Infectious in host (*f_h_*) | 0.012 | 3.34 |
| Disease mortality (*m_d_*) | -0.010 | -2.70 |
| Latency in host (*b_h_*) | 0.006 | 1.75 |
| Dispersal rate (*i*) | 0.004 | 1.25 |
| Latency – soil (*b_s_*) | 0.0002 | 0.06 |

R^2^=0.60, F_12,987_=127.4
